# Comparison of individual hive and apiary-level sample types for spores of *Paenibacillus larvae* in Saskatchewan honey bee operations

**DOI:** 10.1371/journal.pone.0263602

**Published:** 2022-02-07

**Authors:** Michael W. Zabrodski, Jessica E. DeBruyne, Geoff Wilson, Igor Moshynskyy, Mohsen Sharafi, Sarah C. Wood, Ivanna V. Kozii, Jenna Thebeau, Colby D. Klein, Igor Medici de Mattos, LaRhonda Sobchishin, Tasha Epp, Antonio C. Ruzzini, Elemir Simko

**Affiliations:** 1 Department of Veterinary Pathology, Western College of Veterinary Medicine, University of Saskatchewan, Saskatoon, Saskatchewan, Canada; 2 Crops and Irrigation Branch, Ministry of Agriculture, Government of Saskatchewan, Prince Albert, Saskatchewan, Canada; 3 Department of Large Animal Clinical Sciences, Western College of Veterinary Medicine, University of Saskatchewan, Saskatoon, Saskatchewan, Canada; 4 Department of Veterinary Microbiology, Western College of Veterinary Medicine, University of Saskatchewan, Saskatoon, Saskatchewan, Canada; Universidade de São paulo, BRAZIL

## Abstract

Three commercial honey bee operations in Saskatchewan, Canada, with outbreaks of American foulbrood (AFB) and recent or ongoing metaphylactic antibiotic use were intensively sampled to detect spores of *Paenibacillus larvae* during the summer of 2019. Here, we compared spore concentrations in different sample types within individual hives, assessed the surrogacy potential of honey collected from honey supers in place of brood chamber honey or adult bees within hives, and evaluated the ability of pooled, extracted honey to predict the degree of spore contamination identified through individual hive testing. Samples of honey and bees from hives within apiaries with a recent, confirmed case of AFB in a single hive (index apiaries) and apiaries without clinical evidence of AFB (unaffected apiaries), as well as pooled, apiary-level honey samples from end-of-season extraction, were collected and cultured to detect and enumerate spores. Only a few hives were heavily contaminated by spores in any given apiary. All operations were different from one another with regard to both the overall degree of spore contamination across apiaries and the distribution of spores between index apiaries and unaffected apiaries. Within operations, individual hive spore concentrations in unaffected apiaries were significantly different from index apiaries in the brood chamber (BC) honey, honey super (HS) honey, and BC bees of one of three operations. Across all operations, BC honey was best for discriminating index apiaries from unaffected apiaries (p = 0.001), followed by HS honey (p = 0.06), and BC bees (p = 0.398). HS honey positively correlated with both BC honey (r_s_ = 0.76, p < 0.0001) and bees (r_s_ = 0.50, p < 0.0001) and may be useful as a surrogate for either. Spore concentrations in pooled, extracted honey seem to have predictive potential for overall spore contamination within each operation and may have prognostic value in assessing the risk of future AFB outbreaks at the apiary (or operation) level.

## Introduction

American foulbrood (AFB) is a destructive disease of honey bee larvae caused by the gram-positive bacterium, *Paenibacillus larvae* [1]. Depending on its surrounding environment, *P*. *larvae* may exist in one of two distinct forms: either a replicative, vegetative state, or a resilient, infectious endospore (hereafter referred to as spore) [1, 2]. Spores, once ingested by newly hatched and susceptible larvae, undergo germination within the larval midgut into the vegetative form, which replicate prolifically, eventually invading the haemocoel, killing the larva, and undergoing sporulation to produce over a billion new, infectious spores [3–7]. Bacterial spores of *P*. *larvae* are durable, capable of surviving and maintaining infectivity for decades, and able to withstand environmental extremes and common disinfection procedures [8, 9]. Prevention, eradication, and control of AFB are challenging due to the combination of spore resiliency and the dissemination of large numbers of spores throughout a hive by the normal caretaking actions of worker bees [10]. These same characteristics, however, have been useful in the development of diagnostic testing for the presence of spores in different hive- and colony-associated matrices [11]. These include bees, wax, pollen, bottom-board debris, and honey, all of which have been used as a diagnostic tool to determine the clinical status of a hive, or as a predictive tool to determine the risk of disease outbreak [1, 2, 6, 11–33].

Many of these studies have been performed in regions such as Europe and New Zealand, where the use of antimicrobials in beekeeping is prohibited [12, 13, 15–21, 26, 28–31, 34], and are focused predominantly on the collection and testing of matrices from individual hives. Conversely, there are relatively few studies evaluating the distribution of *P*. *larvae* spores within North America, where apiculture is heavily reliant on the sustained use of antimicrobials to prevent clinical outbreaks of AFB [6, 22, 25, 35]. Metaphylactic antimicrobial use against *P*. *larvae* is only successful at eliminating the vegetative state of the bacteria; spores are unaffected by antibiotics and remain infectious [5, 36]. Antimicrobial use is therefore only successful at controlling and preventing clinical signs of disease [33, 36]. As a result, many North American beekeeping operations that incorporate antimicrobial use in their routine management practices assume that there is widespread spore contamination within their hives, fostering continued, indiscriminate antimicrobial treatment to help ensure sustainability and profitability. This practice precludes a true understanding of the distribution and load of spores of *P*. *larvae* within antibiotic-reliant operations, information that could promote more judicious use of antimicrobials through evidence-based decision-making.

The documented emergence of antimicrobial resistance in *P*. *larvae*, along with recent regulatory changes in Canada to strengthen the veterinary oversight of medically important antimicrobial use in animals [37], may have important ramifications for the control of AFB in not only Saskatchewan, but all of North America, as Saskatchewan beekeeping practices are representative of those across much of the continent [22, 37–40]. Without a thorough understanding of the distribution and concentration of *P*. *larvae* spores within its beekeeping operations, the North American beekeeping industry is inadequately prepared to safely reduce its reliance on the metaphylactic use of antibiotics and is at risk of significant economic losses. As such, more work is needed to determine the value of different individual and apiary-level sample types in describing the distribution and degree of contamination of *P*. *larvae* spores within antibiotic-reliant beekeeping operations.

We previously identified clinical outbreaks of American foulbrood in four large-scale, commercial, honey-producing, beekeeping operations across Saskatchewan in the summer of 2019 with recent or ongoing histories of metaphylactic antibiotic use [41]. We reported a description of these outbreaks in the context of relevant management practices as a means of continuing education for Canadian veterinarians, who have only recently become responsible for the prescription of antimicrobials for apiculture [37]. We returned to three of these operations within days of the recognition of clinical signs of AFB to intensively sample individual colonies from the apiaries with a confirmed case of AFB and apiaries without clinical evidence of AFB to quantify contamination by spores of *P*. *larvae*. In addition, pooled honey samples across multiple apiaries were collected from each beekeeper during routine extraction at the end of the honey-producing season for comparative testing. The objectives of this study were: i) to assess *P*. *larvae* spore concentrations from different sample types within individual hives across apiaries of beekeeping operations that had experienced recent outbreaks of AFB; ii) to assess whether honey collected from honey supers could be used as a surrogate for brood chamber honey or adult bees when assessing spore concentrations within a hive; and iii) to assess the ability of pooled honey samples collected during end-of-season extraction to predict the degree of spore contamination identified through individual hive sampling. Through a descriptive and comparative evaluation of different approaches for detecting spores of *P*. *larvae* from these beekeeping operations, we sought to improve our understanding of AFB outbreaks within antibiotic-reliant management systems. The detection of spores in this study was performed with the intent of identifying techniques with potential use as prognostic indicators of future AFB risk, rather than as a means of evaluating the current health status of the investigated hives. The ultimate goal of this study was to evaluate if pooled, extracted honey (i.e., pooled honey from honey supers) could be used for risk assessment of AFB at the apiary or operation level.

## Materials and methods

### Beekeeping operations

In this study, samples were collected from three large-scale, honey-producing, commercial beekeeping operations across Saskatchewan whose management practices are described in greater detail in Zabrodski *et al*. (2020) (S1 Fig). Both here and in this previous study, these commercial beekeeping operations are referred to as operations A, B, and C [41]. All three beekeeping operations are located in central Saskatchewan, Canada (as defined by latitude). In North America, a commercial beekeeping operation refers to beekeeping performed at a very large scale with large numbers of hives across multiple apiaries. The owner(s) of a commercial beekeeping operation rely on hired staff to assist with routine apiary management, and operations may include honey production and/or pollination services. The beekeeping operations sampled in this study ranged in size between 2,700 and 4,000 honey-producing colonies across 45 to 125 apiaries during the summer of 2019 [41]. Here, the term apiary is synonymous with bee yard (a term predominantly used in North America) and is defined as a collection of hives at a single geographical location. Apiaries of commercial beekeeping operations in Saskatchewan each typically contain between 36 and 56 bee colonies.

One operation (operation A) had recently ceased metaphylactic antibiotic use with oxytetracycline, having last treated its colonies approximately three years prior to sample collection described in this study. The remaining two operations (operation B and operation C) had been treating their apiaries with metaphylactic oxytetracycline in an off-label manner [41]. Operation B had been treating with oxytetracycline twice annually (on-label), but only performing a single application per treatment (off-label) instead of the instructed 3 applications per treatment [41]. Operation C, on the other hand, had only been treating with oxytetracycline once annually in the spring (off-label) [41].

As previously described, each outbreak of AFB was considered to be limited to a single colony (hereafter referred to as the index case) within a single apiary [41]. A confirmed diagnosis of AFB within a colony required both the presence of compatible clinical signs (a combination of larval ropiness and/or larval scale) and laboratory isolation of the causative agent from samples of diseased larval tissue taken from the suspect hive [1, 41]. Samples collected by the Provincial Specialist in Apiculture from the index cases in each beekeeping operation were submitted for antimicrobial susceptibility testing using Kirby Bauer disk diffusion at the Animal Health Laboratory (University of Guelph, Guelph, Ontario), and all isolates were confirmed to be susceptible to oxytetracycline [41]. All three operations in this study were contacted throughout 2020 to determine the incidence of AFB disease over the 2019–2020 winter and 2020 honey-producing season.

### Sample collection from individual hives

**Fig 1:**
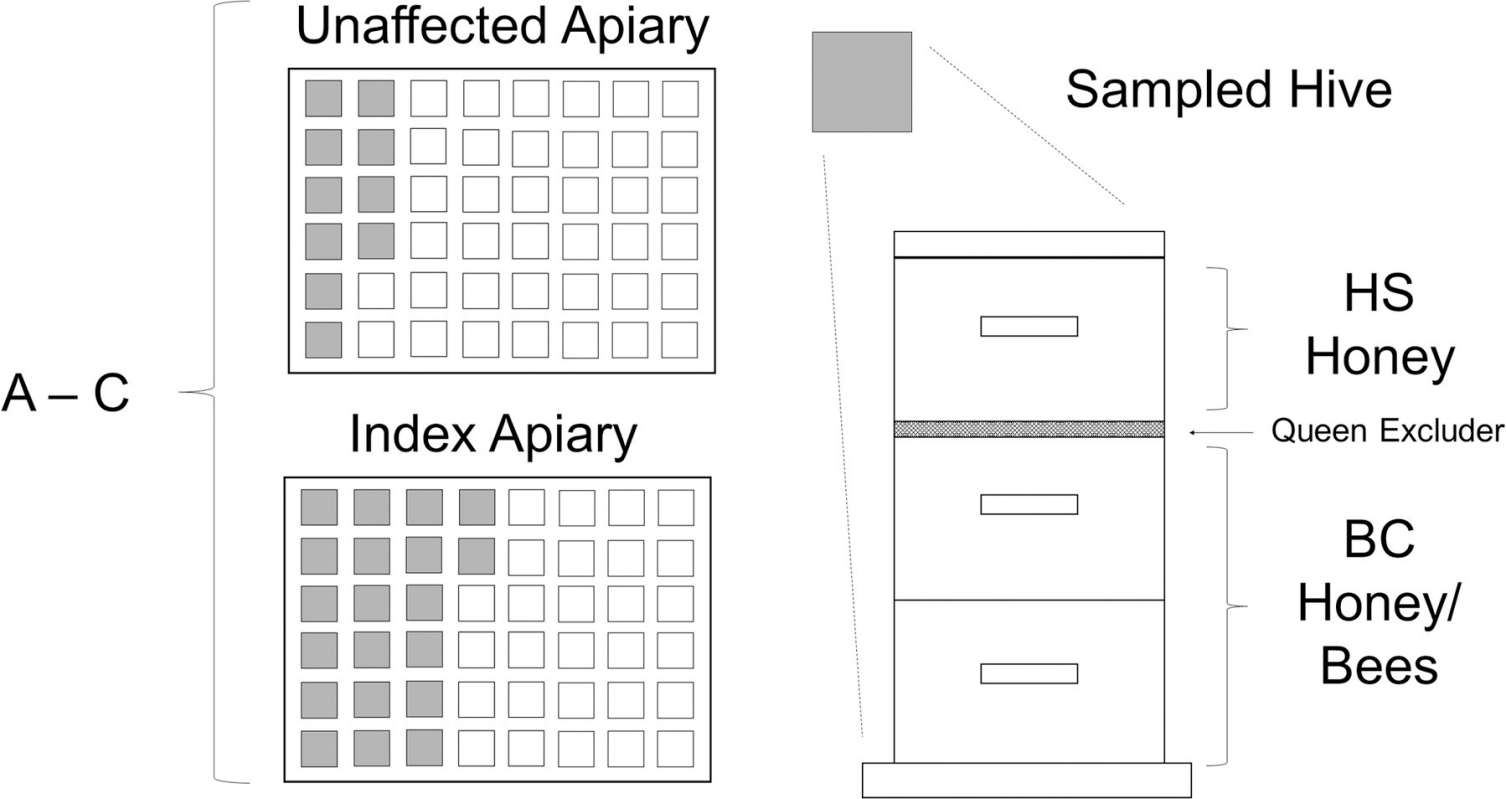
Visual schematic of sampling collection from individual hives. A total of 2 apiaries were sampled for each beekeeping operation (A, B, and C; letters denote operation ID): an apiary with an index case of clinically and laboratory confirmed AFB disease (index apiary) and a second apiary with no detected clinical evidence of AFB disease (unaffected apiary). Twenty randomly selected hives were sampled from the index apiary in each operation (represented by grey-shaded boxes), and ten randomly selected hives were sampled from each unaffected apiary. Samples of honey super honey (HS honey), brood chamber honey (BC honey), and brood chamber bees (BC bees) were collected from each sampled hive, when available.

Following initial reporting of a suspected AFB outbreak (operation A: June 10, 2019; operation B: June 14, 2019; operation C: early June 2019) and subsequent inspection and confirmation by the Provincial Specialist in Apiculture, all three beekeeping operations were subjected to intensive sampling within several days after confirmation (Fig 1). Two apiaries were selected for sampling from each beekeeping operation: the apiary with a recently confirmed case of AFB disease in a single colony (index case), hereafter referred to as index apiary, and a second apiary with no detected clinical evidence of AFB disease, hereafter referred to as unaffected apiary. The second apiary was chosen at the discretion of the beekeeper, with instructions to select a location that was geographically distant from (i.e., not within flying distance of) the index apiary and that they believed to be free of AFB. It should be noted that all sampled colonies in both the index and unaffected apiaries were confirmed to be negative for clinical AFB disease based on the absence of the following clinical signs: a scattered brood appearance, sunken and/or perforated cappings, larval scale against the dependent wall of uncapped cells, and larval ropiness within capped cells [42]. Permission to access each apiary was provided directly by the owner of each beekeeping operation, who was also present during each sampling event.

A total of 30 hives were sampled from each beekeeping operation: 20 hives randomly selected from the index apiary and ten hives randomly selected from the unaffected apiary. The following samples were collected from each hive: i) live, adult bees from a brood chamber frame with uncapped brood (brood chamber bees; hereafter, BC bees); ii) unsealed honey from a brood chamber frame with uncapped brood (brood chamber honey; hereafter, BC honey); and iii) if available, unsealed honey from a frame within the overlying honey super closest to the brood chambers (honey super honey; hereafter, HS honey). If there were no frames of uncapped brood, samples of BC bees and BC honey were collected from frames with or adjacent to capped brood. Samples were collected in individual plastic bags, transported on ice from apiary to laboratory within a few hours after collection, and stored at -20°C until analysis. The Canadian Council on Animal Care (CCAC) does not require permission for research on insects; however, all sampling and animal handling procedures were performed in accordance with the Saskatchewan Apiaries Act.

### Sample collection during end-of-season extraction

To determine if spore concentrations in pooled honey at end-of-season extraction could predict the degree of spore contamination identified through individual hive sampling, beekeepers were instructed to collect a total of 18 honey samples during routine, end-of-season extraction using an adapted, two-stage sampling protocol [43]. For each operation, six apiaries or lots were chosen at the discretion of the beekeeper, and three different samples of honey were collected from each apiary or lot. Here, a lot refers to a collection of several apiaries in close geographic proximity that are collected and extracted together as a single unit during routine honey extraction. Beekeepers selected six different lots or apiaries that were geographically distant from one another. Correlation of these apiaries or lots to the index and unaffected apiaries sampled in June of 2019 was not attempted. Each sample was collected from a different extractor load to ensure that the same frames/hives were not sampled multiple times. Assuming the use of an at least 60-frame commercial extractor and that 60 frames represent between three and six hives within an apiary, the collection of three unique samples from three different extractor loads would represent between nine and 18 hives from a single apiary or lot. Based on this sampling approach, it is assumed that 18 honey samples represented 54 to 108 hives from six geographically distant locations for each operation. Samples were collected and sealed in 500 g plastic tubs and stored at room temperature until processing.

### Preparation of culture media

All samples were cultured on a complex MYPGP medium adapted from previously published protocols [11, 44] with added agonists of *P*. *larvae* spore germination (i.e., L-tyrosine and uric acid [45]). For 1 L of media, 10 g of Difco™ Mueller Hinton Broth (BD, 275730), 15 g of Bacto™ Yeast Extract (BD, 212750), 3 g of potassium phosphate dibasic (Fisher BioReagents, BP363-1), 1 g of sodium pyruvate (Fisher BioReagents, BP356-100), and 15 g of agar (Fisher BioReagents, BP1423-2) were autoclaved in 880 mL of distilled water and combined with 20 mL of separately autoclaved, 10% glucose (Sigma, G-5767). To attain a final volume of 1 L, 50 mL of 60 mM L-tyrosine (Alfa Aesar, A11141) dissolved in 1 M hydrochloric acid (Fisher Chemical, A144-500), 50 mL of 60 mM uric acid (Alfa Aesar, A13346) dissolved in 1 M sodium hydroxide (Fisher Chemical, S318-500), and 1 mL of 20 mg/mL Nalidixic acid (Alfa Aesar, J63550), each sterilized through separate, 0.22 micron filters, were immediately added to the molten media. The resulting MYPGP medium enhanced by germination agonists is hereafter referred to as enhanced MYPGP medium. Plates were refrigerated at 4°C until use.

### Cultivation of *P*. *larvae* from BC bees

A total of 100 worker bees were counted into a plastic bag, mixed with 25 mL of sterile water, and manually crushed according to previously published protocols [15, 19, 21]. A portion of the resulting fluid was heat-treated at 85°C for 15 min [46], allowed to cool to room temperature, and spread onto three plates of enhanced MYPGP media (200 μL per plate, 600 μL total). An additional 200 μL of unpasteurized sample was spread onto a separate plate of enhanced MYPGP media as a control. Samples were briefly vortexed immediately prior to plating. Plates were incubated at 37°C with 5% CO_2_ for seven days [1, 11]. After incubation, the number of bacterial colonies with a morphology consistent with *P*. *larvae* were averaged across the three technical replicates and the number of spores per bee was calculated using the assumption that a single colony forming unit (CFU) is equivalent to a single spore [1, 14]. Serial, ten-fold dilutions were prepared and re-cultured for any samples yielding one or more plates with greater than 100 colonies consistent with *P*. *larvae* morphology to avoid underestimating colony counts due to crowded, confluent colonies [21]. Suspect colonies were submitted to Prairie Diagnostic Services of the Western College of Veterinary Medicine in Saskatoon, Saskatchewan, and were identified as *P*. *larvae* using diagnostic matrix-assisted laser desorption/ionization-time of flight mass spectrometry (MALDI-TOF MS).

### Cultivation of *P*. *larvae* from honey

For BC honey and HS honey collected from individual hives, a mixture ~20 g of honey with accompanying comb wax was mixed with 20 mL of sterile water and shaken overnight at 37°C to ensure complete dissolution of honey [1, 11]. The following morning, samples were filtered through two sheets of loose, autoclaved cheesecloth to remove comb wax and other debris, and the final weight of honey in the suspension was calculated by subtracting the weight of water that had been previously added. Honey suspensions were balanced with additional sterile water and centrifuged at 6,000 *g* for 40 min at room temperature [1, 11] to pellet the spores. The supernatant was poured off and centrifuge tubes left upside-down on paper towel to drain for approximately five min. Pellets were re-suspended in 2 mL of sterile water and the samples vortexed for 20 seconds. Steps for heat treatment, plating, and incubation were identical to those for bee samples.

Samples collected from the extracted, pooled honey contained very little to no wax and did not require straining to remove wax and debris. Accordingly, 20 g from each extracted honey sample was weighed out, mixed with 20 mL of sterile water, and shaken overnight at 37°C. The following morning samples were centrifuged, heat-treated, plated, and incubated as per the steps for honey samples from individual hives. Data for all honey samples were presented as spores per gram of honey.

### ERIC genotyping of *P*. *larvae* isolates

To characterize *P*. *larvae* genotypes isolated from these beekeeping operations, five isolates from each operation (a total of 15 isolates) were subjected to repetitive element PCR fingerprinting (rep-PCR) using ERIC primers [47, 48]. Spore suspensions obtained from both larval scale of the index case and the four BC honey samples with the highest spore concentrations from each operation’s index apiary were revived from -80°C on enhanced MYPGP medium incubated at 37°C with 5% CO_2_ for 72 hours. The resulting populations of *P*. *larvae* colony growth were confirmed as uniform which, as per Bassi *et al* (2015), allows for a single representative colony from each sample to be sub-cultured for rep-PCR [49].

DNA was extracted from sub-cultured isolates using a DNeasy Blood & Tissue kit (Qiagen) following the protocol for gram positive bacteria with minor changes. First, centrifugation of the second buffer was performed at 15,000 rpm rather than 20,000 rpm. Second, 150 μL of elution buffer was used for elution instead of 200 μL.

The DNA sequences used for ERIC primers were as described by Versalovic *et al*. (1994) [47], and the ingredients and reaction cycle parameters for rep-PCR reactions were carried out according to a previously established protocol by Genersch and Otten (2003) [48], but with 40 amplification cycles instead of 35 (Mastercycler™ Pro, manufactured by Eppendorf™, Germany). PCR products were visualized with UV light (302 nm) (AlphaImager HP Imaging System, manufactured by ProteinSimple bio-techne^®^, San Jose, CA, USA) following gel electrophoresis (Bio-Rad) with ethidium bromide run in 1.0% agarose gel at 90 V for 50 min. Differentiation between ERIC I and ERIC II genotypes was determined by the presence or absence of a migrating band between 2500 and 2800 bp that is characteristic of ERIC II [50].

### Data analysis

Statistical analyses were carried out using STATA software (Version 16.1; StataCorp LLC). Descriptive statistics were provided by apiary within beekeeping operation to assess the parameter estimate and its variability (i.e., median and interquartile range) for each individual hive sample type (BC honey, HS honey, BC bees). Normality of data were tested using a Shapiro-Wilk test, and equality of variances assessed with a Levene’s test. Within each operation, the difference between the unaffected and index apiary was compared using a Wilcoxon rank sum test for each sample type. To assess the overall ability of each sample type to discriminate between index and unaffected apiaries, a Poisson regression was used that accounted for operation ID. The robust option was used to estimate the variance–covariance matrix (VCE) corresponding to the parameter estimates, which is robust to some types of misspecification so long as the observations are independent. Correlation between HS honey and BC honey, as well as HS honey and BC bees, was determined using the Spearman rank correlation. A Kruskal-Wallis test was used to compare pooled, extracted honey samples between beekeeping operations. To evaluate pooled, extracted honey samples as a predictor of results of individual hive sampling, all pooled samples within each beekeeping operations were compared to an arbitrary threshold set at 1 spore per gram of honey. An α cut-off of 0.05 was used for all statistical analyses with the exception of post-hoc pairwise comparisons following Kruskal-Wallis assessment of pooled, extracted honey samples between operations, where an α cut-off of 0.017 was used following Bonferroni correction.

## Results

### Descriptive evaluation of *P*. *larvae* spore concentrations in individual hives within apiaries

**Fig 2:**
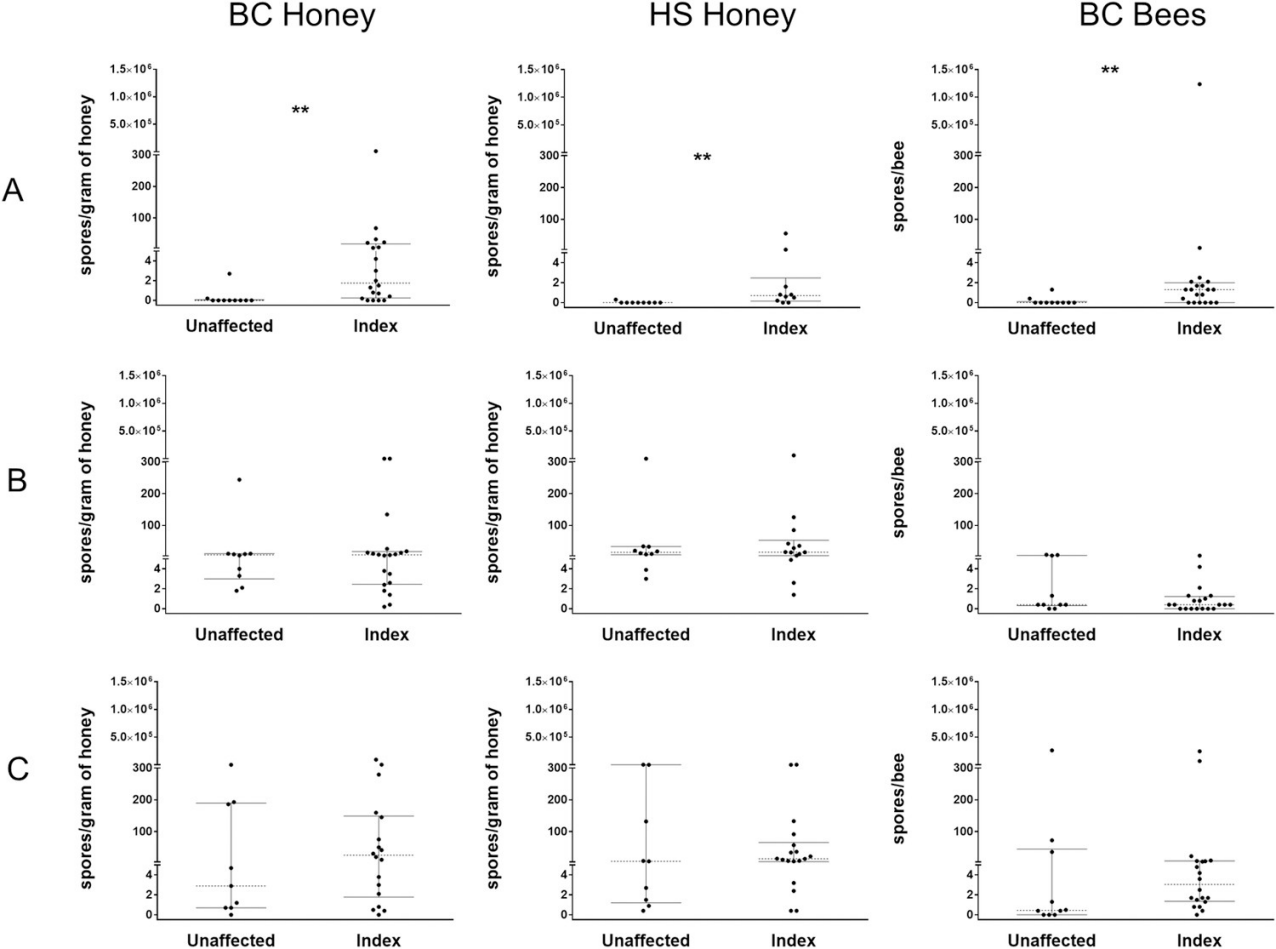
Concentrations of *Paenibacillus larvae* spores for individual hives from index and unaffected apiaries. A single index apiary and single unaffected apiary were sampled from each operation (six apiaries total). Apiaries are grouped vertically by sample type (BC honey, HS honey, and BC bees) and horizontally by beekeeping operation (A, B, and C; letters denote operation ID). Dotted lines represent median values and bars represent interquartile ranges. ** denotes statistical significance where p < 0.01 (Wilcoxon rank sum test).

In all operations, a small number of hives within apiaries accounted for the majority of spore contamination. Variability in spore concentrations between hives within individual apiaries was dependent on the beekeeping operation (Table 1, Fig 2, S1 Dataset). In operation A, spore concentrations within the unaffected apiary were low and tightly clustered regardless of sample type. Within the index apiary, a relatively small number of samples within each sample type accounted for the majority of spore contamination. A single BC honey sample (1/20) and a single bee sample (1/20) from the index apiary had spore concentrations greater than 100 spores/g or spores/bee, respectively.

**Table 1:** Summary statistics of concentrations of *Paenibacillus larvae* spores from individual hives.

		Unaffected apiary			Index apiary		
Operation ID		BC honey (spores/g)	HS honey (spores/g)	BC bees (spores/bee)	BC honey (spores/g)	HS honey (spores/g)	BC bees (spores/bee)
A	Sample size	10	9	10	20	10	20
	Median	0.0	0.0	0.0	1.8	0.7	1.3
	Minimum	0.0	0.0	0.0	0.0	0.0	0.0
	Maximum	2.7	0.3	1.3	10,703.2	56.0	1,237,500.0
	Interquartile range	0.0	0.0	0.0	13.9	1.4	1.9
B	Sample size	10	10	10	20	14	20
	Median	7.9	15.8	0.4	8.1	16.6	0.4
	Minimum	1.8	3.0	0.0	0.2	1.4	0.0
	Maximum	243.8	747.6	8.3	670.0	61,666.7	5.4
	Interquartile range	8.2	24.4	5.4	14.6	37.6	1.2
C	Sample size	9	9	10	18	18	20
	Median	2.9	6.9	0.5	25.8	14.9	3.0
	Minimum	0.0	0.4	0.0	0.0	0.4	0.0
	Maximum	1,163.6	2,684.9	262,500.0	94,589.5	3,574.1	245,833.3
	Interquartile range	185.2	130.4	36.2	143.2	50.8	6.2

Summary statistics of concentrations of *Paenibacillus larvae* spores from individual hives for honey collected from brood chambers (BC Honey) and honey supers (HS Honey) and bees collected from brood chambers (BC Bees) within apiaries with a recent, confirmed case of American foulbrood (AFB) in a single hive (index apiaries) and apiaries unaffected by AFB (unaffected apiaries). Capital letters denote operation ID (A, B, or C).

For operation B, variability of spore concentrations between hives across all sample types in the unaffected apiary was relatively comparable to that of the index apiary. One to three samples from each sample type were responsible for the majority of spore contamination within each apiary. One BC honey sample (1/10) and one HS honey sample (1/10) from the unaffected apiary had spore concentrations greater than 100 spores/g. Three BC honey samples (3/20) and two HS honey samples (2/14) from the index apiary had spore concentrations greater than 100 spores/g.

In operation C, the distribution of spore concentrations between hives across all sample types in the unaffected and index apiary were similar, and overall had a greater number of highly contaminated samples in each sample type relative to other operations. Three samples of BC honey (3/9), three HS honey samples (3/9), and one bee sample (1/10) had spore concentrations greater than 100 spores/g or spores/bee in the unaffected apiary. In the index apiary, five BC honey samples (5/18), three HS honey samples (3/18), and two bee samples (2/20) had spore concentrations greater than 100 spores/g or spores/bee.

### Comparison of *P*. *larvae* spore concentrations in individual hives between index and unaffected apiaries

With regard to the overall discriminating ability of each individual hive sample type, only BC honey was able to detect a difference between index and unaffected apiaries (after accounting for operation ID) (Robust Coefficient [RC] = 3.39, 95% Confidence Interval [CI] = 1.36 to 5.41, p = 0.001; Poisson regression). No statistically significant difference was detected between index and unaffected apiaries (after accounting for operation ID) when measuring spore concentrations from either HS honey (RC = 2.14, 95% CI = -0.091 to 4.36, p = 0.06; Poisson regression) or BC bees (RC = 1.08, 95% CI = -1.43 to 3.59, p = 0.398; Poisson regression).

Within each operation, the difference between index and unaffected apiaries was dependent on the operation ID and the sample type used for assessment (Fig 2). Within operation A, statistical differences in spore concentrations between index and unaffected apiaries were observed for BC honey (z = 3.115, p = 0.0018; Wilcoxon rank sum test), HS honey (z = 3.003, p = 0.0027; Wilcoxon rank sum test), and BC bees (z = 2.723, p = 0.0065; Wilcoxon rank sum test). No significant differences were found between the index and unaffected apiaries within operations B and C by any of the individual hive sample types.

### Comparison of *P*. *larvae* spore concentrations in individual hive HS honey to BC honey and BC bees

**Fig 3:**
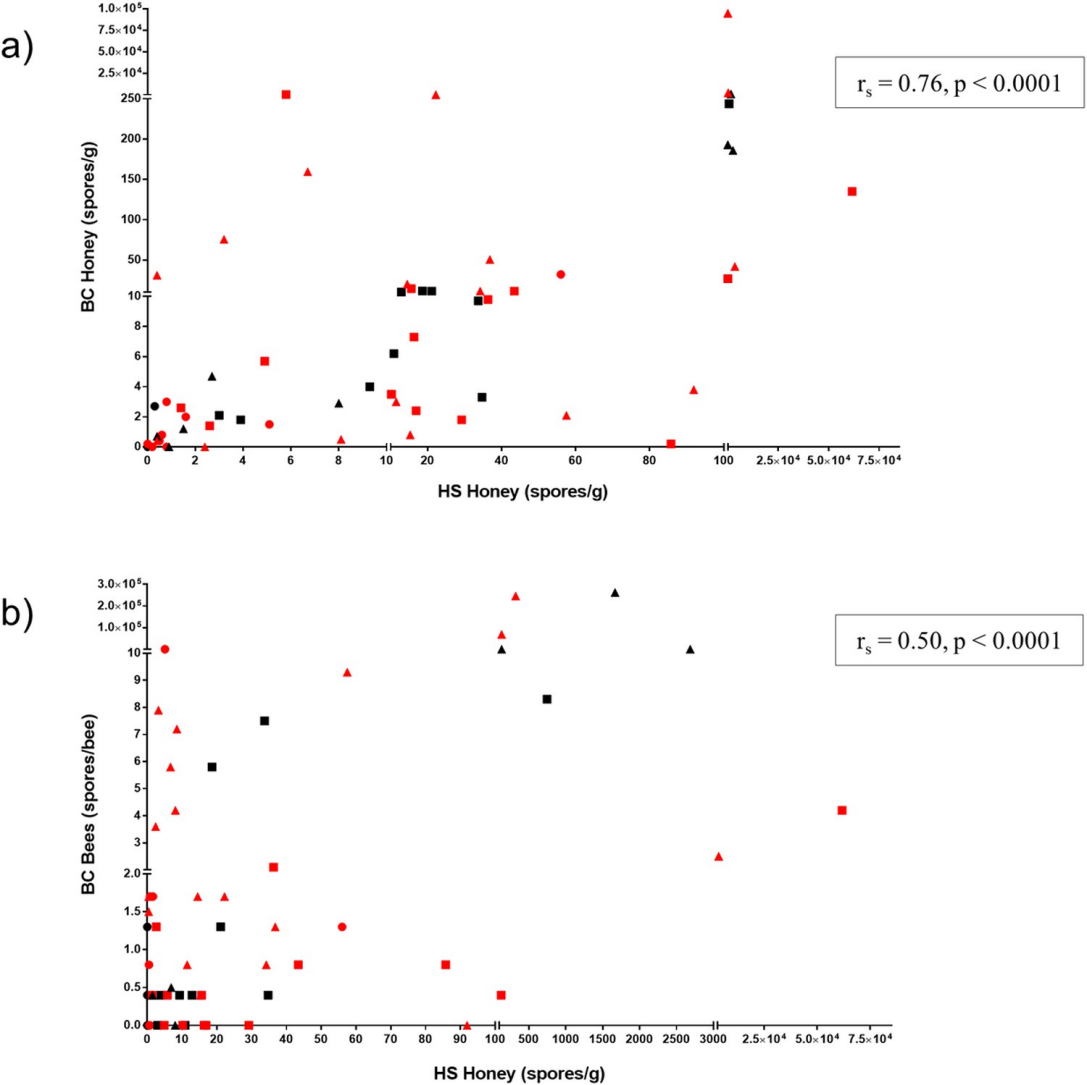
Comparison of concentrations of *Paenibacillus larvae* spores between HS honey and BC honey and BC bees within individual hives. Data include all apiaries of all beekeeping operations. a) HS honey is positively correlated with BC honey (r_s_ = 0.76, p < 0.0001); b) HS honey is positively correlated with BC bees, albeit less strongly (r_s_ = 0.50, p < 0.0001). Hives with only one of the two samples under comparison are excluded from each plot. Circles = operation A, squares = operation B, triangles = operation C; black = hive from apiary unaffected by American foulbrood (AFB), red = hive from index apiary with a recent, confirmed case of AFB in a single hive.

HS honey was assessed as a surrogate for BC honey and BC bees in measuring spores of *P*. *larvae* by correlating these values in individual hives. HS honey was positively correlated with BC honey (r_s_ = 0.76, p < 0.0001, Fig 3A). Similarly, HS honey was positively correlated with BC bees, albeit less strongly (r_s_ = 0.50, p < 0.0001, Fig 3B). Hives with only one of the two samples under comparison were excluded from analysis, resulting in 68 pairwise observations for HS honey and BC honey, and 70 observations for HS honey and BC bees.

In operation A, 19 out of possible 30 hives from across both apiaries had honey samples available from both the honey super and brood chamber at the time of sampling. Of these, ten hives (10/19) had detectable spores in at least one of either HS honey or BC honey. Spore concentrations from BC honey samples were higher than those from honey supers in five of these hives (5/10). Twenty-four of 30 hives across both apiaries in operation B had available honey from both the honey super and brood chamber. All 24 of these hives had detectable spores in at least one of their honey samples, and spore concentrations from BC honey were higher than those from honey supers in three hives (3/24). In operation C, 25 out of a possible 30 hives from across both apiaries had honey samples available from both the honey super and brood chamber. All 25 of these hives had detectable spores in at least one of their honey samples, and spore concentrations from BC honey samples were higher than or equal to those from honey supers in 12 hives (12/25).

The majority of bee samples across all apiaries of all beekeeping operation (82/90) had low levels of detectable spores with fewer than 10 spores per bee (Range = 0–9.3 spores/bee). Five of the eight hives with bee samples greater than 10 spores per bee had concurrently high concentrations of detectable spores in HS honey (Range = 131.9–2,684.9 spores/g). Of the remaining three hives with relatively high spore concentrations in BC bees, two had no available HS honey, whereas the third (10.8 spores/bee) detected a maximum of 5.1 spores per gram of honey in its super honey sample.

### Apiary-level sampling for spores of *P*. *larvae* in pooled, extracted honey and subsequent incidence of AFB

**Fig 4:**
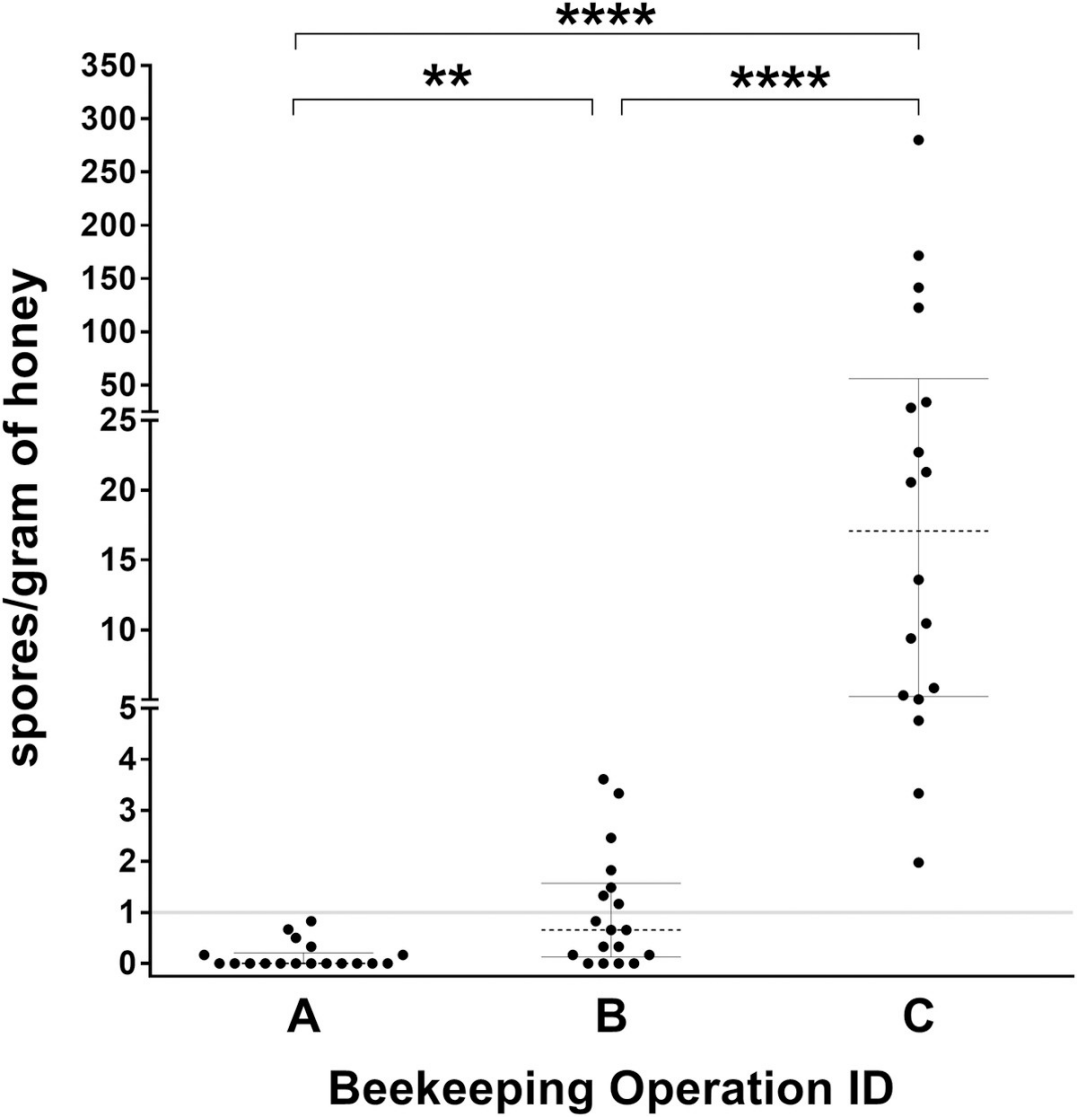
Spores of *Paenibacillus larvae* per gram of honey from pooled honey samples collected during routine extraction at the end of the honey-producing season for each beekeeping operation. Each operation submitted 18 samples representing six randomly selected apiaries or lots; three unique samples were collected from each apiary or lot. Dotted lines represent median values and bars represent interquartile ranges. The solid grey line represents an arbitrary threshold value of one spore per gram of honey. Capital letters denote operation ID. ** denotes statistical significance where p < 0.01; **** denotes statistical significance where p < 0.0001 (Kruskal-Wallis test).

Spore concentrations of extracted honey samples from all operations were different from one another (Fig 4). Operation A had the lowest concentration of detectable spores per gram of honey across its pooled samples (M = 0, Range = 0 to 0.8, IQR = 0.2), followed by increasing concentrations of detectable spores in operation B (M = 0.7, Range = 0 to 3.6, IQR = 1.3), and operation C (M = 17.1, Range = 2.0 to 280.3, IQR = 28.8). When compared to an arbitrary threshold of 1 spore per gram of honey, 0% of operation A’s 18 pooled samples (0/18), 39% of operation B’s pooled samples (7/18), and 100% of operation C’s samples (18/18) fell above this threshold line.

Median spore concentrations were assessed as a rough assessment of the ability for pooled honey samples to reflect the degree of contamination detected by individual HS honey samples across apiaries within an operation. The median spore concentration of all individual hive HS honey samples from Operation A was 0 spores/g, which was comparable to the median spore concentration from pooled honey samples (0 spores/g). The median spore concentration of all HS honey samples from Operation B was 16.6 spores/g, whereas the median spore concentration from the pooled samples was 0.7 spores/g. The median spore concentration of all HS honey samples from Operation C was 11.5 spores/g and the median concentration from the pooled samples was 17.1 spores/g.

Both operation A and operation B reported no further cases of AFB throughout 2020. Operation C reported clinical signs consistent with AFB within several colonies that died overwinter (2019/20), which were confirmed by the provincial specialist in apiculture. These colonies belonged to apiaries that had not been included in either individual or apiary-level sampling during 2019.

### ERIC genotyping of *P*. *larvae* isolates

The banding patterns of the ERIC-PCR products of all 15 isolates across all beekeeping operations were uniform and consistent with ERIC I (S2 Fig). These patterns consisted of a 970 bp migrating band and the absence of a migrating band between 2500 and 2800 bp [50]. No isolates were compatible with ERIC II.

## Discussion

Through the opportunistic and intensive sampling of beekeeping operations with recent clinical outbreaks of AFB, we found that pooled samples of HS honey collected during end of season extraction were reflective of the overall severity of contamination by AFB spores within an operation and may have potential utility as a prognostic indicator of AFB risk. In addition, we demonstrated that only a few hives were heavily contaminated with spores and most hives had few to no detectable spores in any given apiary. Index apiaries tended to have higher concentrations of spores than unaffected apiaries, but this was largely dependent on sample type and the beekeeping operation under examination. In the context of overall discriminatory ability, BC honey was best for differentiating between index and unaffected apiaries, although HS honey was strongly correlated with BC honey and may be used as a surrogate in place of brood chamber samples.

Similar to studies performed in Europe, where antibiotic use in apiculture is prohibited [51, 52], we found that spores are not homogenously distributed amongst hives within apiaries treated with antibiotics, and apiary contamination with spores appears to be driven by only a few heavily contaminated hives. This is contrary to our initial expectation that hives within an apiary would have comparable levels of detectable spores, as previous work has shown that the introduction of spore-laden, recently extracted (wet) honey supers onto some AFB-free hives within an AFB-free apiary results in rapid dissemination of spores to bees from all hives [30]. Similarly, research evaluating the impact of robbing behaviour on the horizontal transmission of *P*. *larvae* spores found that hives within close proximity of clinically diseased hives were at a high risk of contracting high levels of spores [53].

**Fig 5:**
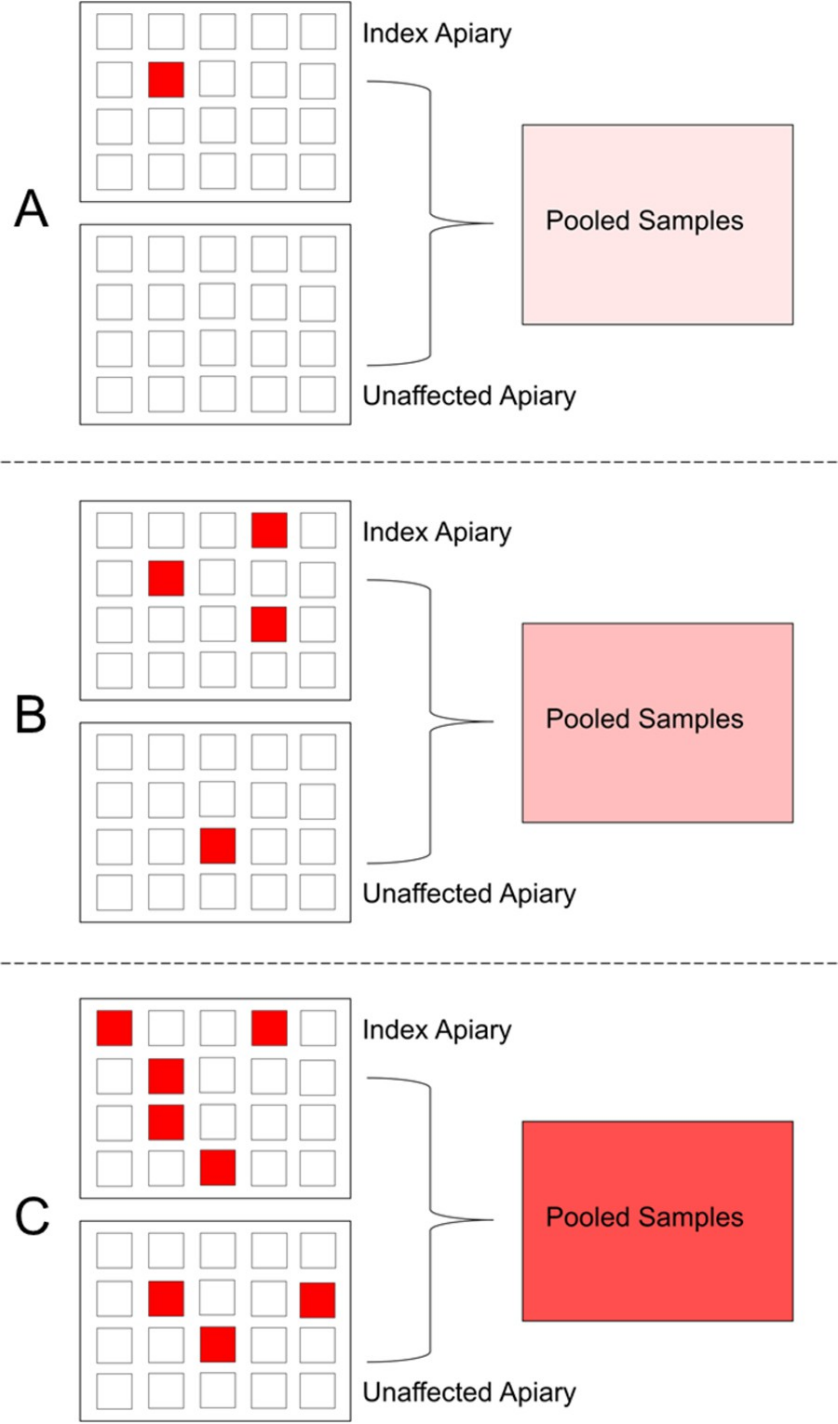
Conceptual representation of the detection of a spore “signal” in pooled, apiary-level honey samples. The corresponding apiaries the pooled samples are derived from are indicated by letters for operations A, B, and C. Small boxes within an apiary represent individual bee hives, and red boxes represent those hives heavily contaminated with spores of *P*. *larvae*. A lighter “signal” (light pink/red) in the pooled samples reflects operations with very little contamination in individual hives, whereas higher concentrations of spores (stronger “signal”–red) in pooled samples correspond to more widespread and/or chronic contamination. Index and unaffected apiaries are used in this theoretical example.

Although BC honey had a better overall ability to discriminate between index and unaffected apiaries relative to HS honey based on the detection of spores, we found that HS honey was positively correlated with spore concentrations in both BC honey and BC bees. Furthermore, the overall burden of spores identified within each operation through pooled, extracted honey from honey supers was comparable to the severity of contamination identified in individually sampled hives (Fig 5). We therefore suggest that HS honey may be used as a surrogate for brood chamber sample types as a means of identifying spore concentrations within hives and that the use of pooled HS represented in extracted samples may have a couple of distinct advantages over the use of BC bees and BC honey in large, antibiotic-reliant beekeeping operations.

First, HS honey can be easily scaled-up into pooled samples representing multiple hives within an apiary by collecting during routine honey extraction at the end of a honey-producing season. The main advantage of this sampling approach is in its relative convenience to the beekeeping operation, as honey samples may be rapidly collected during the spinning of frames on an extractor with minimal disturbance to normal workflow [22, 32]. Due to the skewed distribution of spore contamination within an apiary, a large sample size is required on a per apiary basis to identify the relatively few, heavily contaminated, and presumably high-risk hives. The sampling of individual hives is therefore time consuming, laborious, and logistically impossible for large-scale North American beekeeping operations. Collecting several samples of honey from separate extractor loads incorporates multiple hives at a time with a higher chance of “capturing” the few, heavily contaminated hives that may be present within an apiary.

Second, the detection of a spore “signal” through apiary-level testing may reflect the overall chronicity and severity of *P*. *larvae* spore contamination across an entire operation, despite use of antibiotic metaphylaxis (Fig 5) [22]. At the very least, this approach has merit in its ability to screen apiaries within a beekeeping operation for contamination level by *P*. *larvae* spores that may signal the need for closer investigation on a per hive basis, similar to work done in other studies [22, 26, 32, 34, 35]. In this study, where a limited number of operations are examined, AFB re-occurred in the most contaminated operation (operation C) within 12 months, suggesting that spore concentrations in pooled, extracted honey may be predictive of the risk of clinical AFB, as suggested in other studies performed in regions where antibiotic use in apiculture is prohibited [26, 34]. Accordingly, we are expanding the scope of this investigation to test the predictive value of pooled honey across a large number of commercial beekeeping operations reliant on chronic antibiotic use in the control of AFB.

Finally, honey samples in this study had a more gradated distribution of spore concentrations relative to those in bees, which were either very low or very high. Honey samples, within the context of these operations, may therefore have greater utility as a predictor of AFB risk due to an increased ability to discern low, medium, and high concentrations, which may in turn correspond to low, moderate, and high categories of AFB risk. This idea is supported by Von der Ohe and Dustmann (1997), who used spore concentrations in honey to establish contamination classes that corresponded to the risk of developing AFB disease [26]. Similarly, Hansen and Rasmussen (1986) demonstrated that spore concentrations in pooled honey samples had predictive potential for the development of clinical signs of AFB in the following year [34]. Verification of such risk categories, however, would require longitudinal observations for changes in spore concentrations within these hives, as well as the reporting of any emergence of AFB, which we are currently investigating.

In contrast to the gradated distribution of results in honey, the either very low or very high results for bee samples in this study suggest that bees may be better suited as an indicator of current colony health status, as has been demonstrated by previous studies [14, 15, 18, 19, 21, 31, 51]. There are several factors that, taken together with antimicrobial use, may account for the either very low or very high results observed. First, spores of *P*. *larvae* are not uniformly distributed amongst bees, and only a small proportion of bees within a contaminated hive will carry the majority of spores [18]. Second, the proportion of spore-positive bees within a hive is positively correlated to the severity of active infection [18]. Third, spore abundance within bees is also positively correlated to the severity of active infection [14, 20, 22, 51]. Erban *et al*. (2017), when comparing the abundance of *P*. *larvae* spores within the microbiome of honey bees sampled from colonies with clinical signs of AFB to those from adjacent, asymptomatic colonies and distant, asymptomatic colonies, found increased abundance of *P*. *larvae* in clinically affected colonies only [51]. Unaffected colonies in close proximity to those with clinical signs of AFB were found to be no different from those outside of the designated AFB zone [51]. In beekeeping operations with a chronic reliance on metaphylactic antibiotic use to prevent and control clinical signs of AFB, there is expected to be very little evidence of active disease. This may, in turn, reduce the overall number of bees with high spore burdens, thereby reducing the likelihood of sampling these relatively few individuals. This idea may be indirectly supported by a study assessing the use of bees and bulk honey samples as a means to detect *P*. *larvae* spores in Manitoba beekeeping operations, whose management practices are comparable to those in Saskatchewan [22]. Pernal and Melathopoulos (2006) observed that bee samples yielded no *P*. *larvae* in some operations with histories of chronic, recurrent, and current AFB infection [22].

This study has several important limitations. First, as a cross-sectional, opportunistic survey, this study lacks longitudinal assessment of the individually sampled hives. Second, the sampled operations ranged in size between approximately 2,700 and 4,000 honey-producing hives distributed amongst anywhere between 45 and 125 separate bee apiaries. Sampling in this study was limited to 30 individual hives (0.8–1.1% of total honey-producing hives) and two apiaries (1.6%– 4.4% of total apiaries) per operation. This small selection may not reflect the entire operation as a whole, however, the results from individually sampled hives positively correlate with the corresponding degree of spore contamination identified within the pooled, apiary-level honey samples, which we estimate are representative of between 54 and 108 hives from across each operation. Third, there are inherent limitations to the plate culturing technique used in this study. It is recognized that our collective, overall ability to cultivate vegetative *P*. *larvae* from spores on different artificial media is relatively poor [11]. Not all spores will readily germinate, and previous studies have determined that fewer than 10 percent of *P*. *larvae* spores within a sample will germinate and produce visible colony growth [24, 54]. The spore concentrations reported in this study may therefore underestimate the true number of spores present within the sampled hives and pooled honey samples. While this may be the case, all operations would be equally affected by suboptimal germination rates and would therefore not affect the comparative AFB risk assessment. Finally, in addition to poor overall germination, protocols for the cultivation of *P*. *larvae* spores are biased toward the detection of ERIC I strains over ERIC II strains [55]. Heat treatment, which is a necessary step to eliminate contaminate overgrowth, stimulates the germination of ERIC I strains at temperatures over 90°C while inhibiting the germination of ERIC II [55]. The protocols used in this study were designed to be permissive to the growth of any ERIC II strains through the use of a heat treatment that did not exceed 85°C and through the simultaneous culture of suspension not subjected to heat. Despite this, all isolates tested across all beekeeping operations, including those obtained from each index case, were identified as ERIC I. The exclusive recognition of ERIC I strains in these three beekeeping operations is consistent with our limited understanding of the prevalence of ERIC genotypes in the Americas [56].

Surprisingly, of the three operations asked to select an unaffected apiary free of AFB, only operation A was successful in identifying an apiary that concurrently had a relatively low degree of spore contamination. Although the unaffected apiaries in both operation B and operation C were free of clinical signs of disease, they still had individual hives with levels of spore contamination comparable to those in the index apiaries. Based upon spore thresholds for clinical AFB in other studies [14, 52], it is reasonable to expect that very high concentrations of spores in these seemingly healthy hives would likely be associated with the presence of clinical/subclinical AFB if not for the recent and/or continual application of antibiotic therapy. This raises concern that beekeeping operations, through the chronic use of antibiotic metaphylaxis, may underestimate the severity of contamination within their operations and could potentially benefit from convenient, apiary-level testing of pooled honey, as identified in this study. This would allow beekeeping operations to identify those apiaries at risk of AFB outbreaks, allowing them to implement targeted interventions to mitigate risk and make evidence-based decisions regarding the use of antibiotics.

In conclusion, by comparing BC bees, BC honey, and HS honey within individual hives, we have demonstrated the usefulness of HS honey as a surrogate for both BC bees and BC honey. In addition, we have shown that pooled samples of HS honey collected during routine extraction are reflective of the overall degree of spore contamination within a given operation and may potentially be used as a prognostic indicator of the risk of future AFB outbreak. These findings improve our understanding of AFB outbreaks within antibiotic-reliant management systems and provide a potential avenue for the development of prognostic testing for AFB risk through pooled, apiary-level honey that will help to establish meaningful surveillance data for commercial beekeepers in North America.

## Supporting information

S1 Dataset Raw data of concentrations of *Paenibacillus larvae* spores for individual hives from index apiaries with a recent, single case of American foulbrood (AFB) and apiaries unaffected by AFB. (XLSX)

S1 Fig Schematic comparing previous case report (Zabrodski *et al*. 2020) and current study. (TIF)

S2 Fig Gel electrophoresis patterns for *Paenibacillus larvae* genotyping using rep-PCR with ERIC primers. Lanes 1 and 17 contain N0550A and N0468S Quick-Load^®^ DNA ladders, respectively. Lanes 2, 4, 5, 6, and 7 contain isolates from operation A; lanes 8 through 12 contain isolates from operation B; lanes 3 and 13 through 16 contains isolates from operation C. All patterns include a 970 bp migrating band and the absence of a migrating band between 2500 and 2800 bp. Differentiation between ERIC I and ERIC II genotypes was determined by the presence or absence of a migrating band between 2500 and 2800 bp that is characteristic of ERIC II.
(TIF)
