# Supplementary figures and images for: Comparison of individual hive and apiary-level sample types for spores of Paenibacillus larvae in Saskatchewan honey bee operations
Source: PLoS One. 2022 Feb 7;17(2):e0263602. doi: 10.1371/journal.pone.0263602 (PMC8820611; doi:10.1371/journal.pone.0263602)

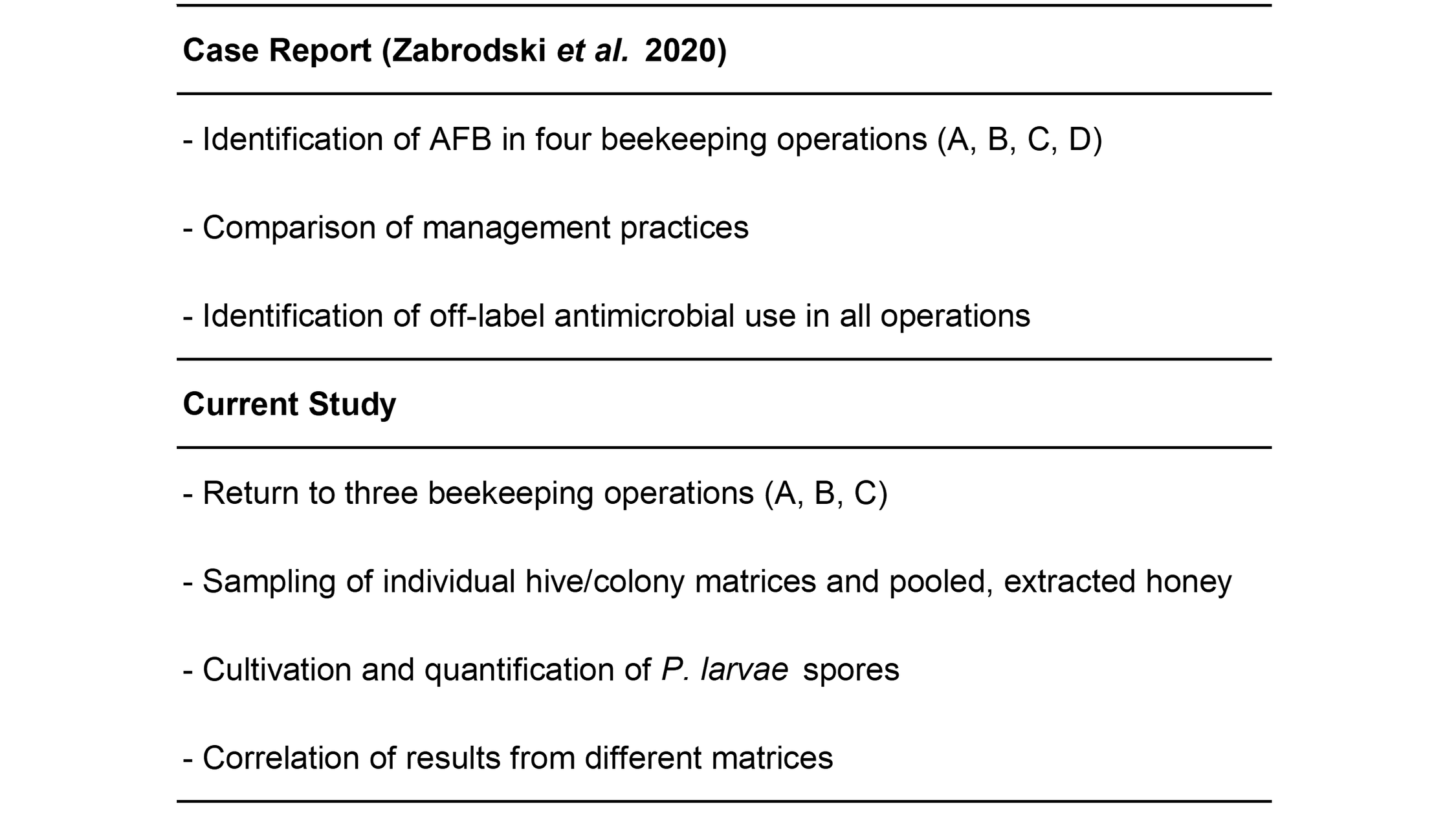

Supplement: S1 Fig — (TIF) [file pone.0263602.s002.tif]

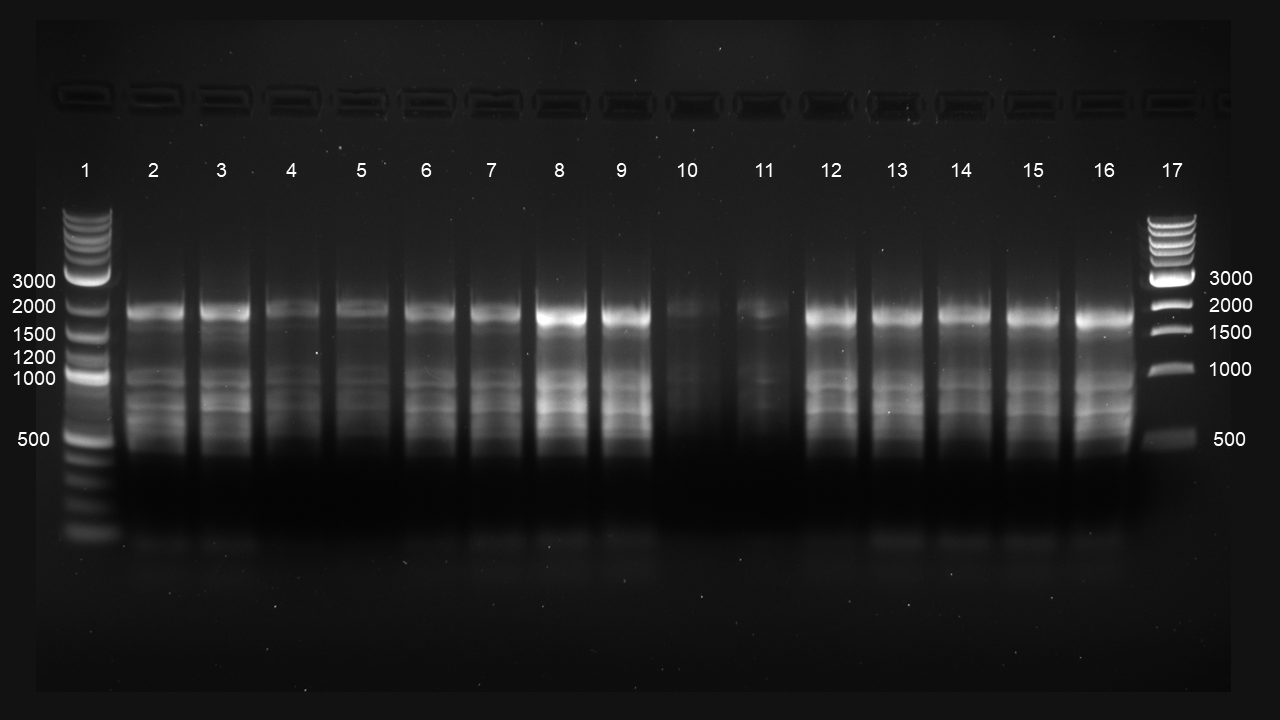

Supplement: S2 Fig — Lanes 1 and 17 contain N0550A and N0468S Quick-Load® DNA ladders, respectively. Lanes 2, 4, 5, 6, and 7 contain isolates from operation A; lanes 8 through 12 contain isolates from operation B; lanes 3 and 13 through 16 contains isolates from operation C. All patterns include a 970 bp migrating band and the absence of a migrating band between 2500 and 2800 bp. Differentiation between ERIC I and ERIC II genotypes was determined by the presence or absence of a migrating band between 2500 and 2800 bp that is characteristic of ERIC II. (TIF) [file pone.0263602.s003.tif]
